# A Critical Review on Hygrothermal and Sound Absorption Behavior of Natural-Fiber-Reinforced Polymer Composites

**DOI:** 10.3390/polym14214727

**Published:** 2022-11-04

**Authors:** V. Bhuvaneswari, Balaji Devarajan, B. Arulmurugan, R. Mahendran, S. Rajkumar, Shubham Sharma, Kuwar Mausam, Changhe Li, Elsayed Tag Eldin

**Affiliations:** 1 Department of Mechanical Engineering, KPR Institute of Engineering and Technology, Arasur 641407, India; 2 Department of Mechanical Engineering, Jai Shriram Engineering College, Avinashipalayam 638660, India; 3 Department of Mechanical Engineering, Faculty of Manufacturing, Institute of Technology, Hawassa University, Awasa 3870006, Ethiopia; 4 University Centre of Research and Development, Mechanical Engineering Department, Chandigarh University, Mohali 140413, India; 5 School of Mechanical and Automotive Engineering, Qingdao University of Technology, Qingdao 266520, China; 6 Department of Mechanical Engineering, GLA University, Mathura 281406, India; 7 Faculty of Engineering and Technology, Future University in Egypt, New Cairo 11835, Egypt

**Keywords:** natural fiber, polymer matrix, bio-composites, moisture absorption, sound absorption

## Abstract

Increasing global environmental problems and awareness towards the utilization of eco-friendly resources enhanced the progress of research towards the development of next-generation biodegradable and environmentally friendly material. The development of natural-based composite material has led to various advantages such as a reduction in greenhouse gases and carbon footprints. In spite of the various advantages obtained from green materials, there are also a few disadvantages, such as poor interfacial compatibility between the polymer matrix and natural reinforcements and the high hydrophilicity of composites due to the reinforcement of hydrophilic natural fibers. This review focuses on various moisture-absorbing and sound-absorbing natural fiber polymer composites along with the synopsis of preparation methods of natural fiber polymer composites. It was stated in various studies that natural fibers are durable with a long life but their moisture absorption behavior depends on various factors. Such natural fibers possess different moisture absorption behavior rates and different moisture absorption behavior. The conversion of hydrophilic fibers into hydrophobic is deemed very important in improving the mechanical, thermal, and physical properties of the natural-fiber-reinforced polymer composites. One more physical property that requires the involvement of natural fibers in place of synthetic fibers is the sound absorption behavior. Various researchers have made experiments using natural-fiber-reinforced polymer composites as sound-absorbing materials. It was found from various studies that composites with higher thickness, porosity, and density behaved as better sound-absorbing materials.

## 1. Introduction

Reducing the use of fossil fuels, particularly in the construction industry, is a major focus of scientific investigation. Buildings accounted for 21% of global final energy consumption in 2017, placing them third behind industry and transportation. Global final consumption energy, as well as unused, dissipated at a rate of 9.1% [1]. Energy consumption in buildings has enhanced over the last few decades, which necessitates more power generation and, consequently, CO_2_ emissions. Climate policy has undergone radical transformations in the last three decades, particularly since the United Nations’ publication [2] in 1987, also recognized as the Brundtland Report. One of the most important aspects of sustainable development is protecting the environment and climate, and also bringing the global environment and development issues into the official concerns of all nations. With this as a backdrop, the UNFCCC [3] came into being as a means of addressing both global climate change and economic growth, particularly in energy-intensive industries such as construction. Ecosystem stability and efficiency are therefore the focus of numerous efforts at the global and national levels, rather than those made in Europe.

Categorizations of international conventions and protocols, including the 1987 Montréal and Kyoto Protocols, as well as the 1997 editions [4,5] of both, and the 2016 Paris agreement [6], were developed to classify these endeavors. One of their objectives is to maintain greenhouse gas levels at a level that protects biodiversity as well as enabling the environment to participate in climate change in a traditional manner. Buildings seem to be Europe’s second-largest energy consumer, consuming up to 26.1% of the EU’s energy consumption [7]. A few new trends are currently being selected to minimize elevated greenhouse gas emissions as well as minimize energy use. Building heating energy accounts for approximately 63.6% [8] of the residential building sector’s overall energy consumption. As a result, two major trends have emerged in recent years to combat rising energy consumption as well as greenhouse gas emissions: improving building structure envelope insulation efficiency to minimize energy consumption as well as developing new low carbon materials, and reducing the carbon dioxide footprint. It is possible to achieve these trends through the use of porous materials such as concrete [9,10], fibers concrete [11,12,13,14,15], bio-based material [16,17] as natural fiber components, straw and rammed earth. In comparison to denser concrete, the lower thermal conductivity that these porous materials achieve is the primary benefit of their use.

**Figure 1:**
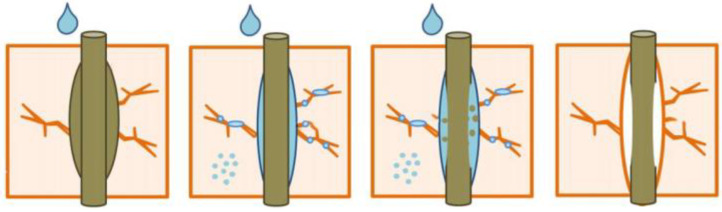
Degradation of composite interface due to moisture absorption (Adapted from the reference [4]).

Although mechanical strength decreases with porousness, an acceptable balance must be accomplished between mechanical and thermal properties. Buildings have the ability to trap moisture as well as potentially relieve it depending on climate and outside conditions when this mechanical thermal stability is met, due to their greater porosity and tortuosity. They must be considered when measuring and simulating the entire building’s power requirements [18]. Regulations and labeling, as well as decision-making, are made more difficult by effective power simulation of this type of building construction. We currently lack a reference model for this envelope type’s transient hygrothermal behavior. Furthermore, no model has been developed yet to account for the influence of coupled heat and mass transfer concepts on the world’s energy efficiency of this construction type. Coupling heat and moisture transfers of construction elements, as well as envelopes, have become increasingly important for simulation methods in the past few decades [19]. Figure 1 shows the schematic of the degradation of the composites’ interface due to moisture absorption. It clearly portrays the absorption of water molecules into the voids present in between the fiber–matrix interface and the development of matrix cracks due to excessive water absorption.

Further research into potential low-carbon buildings’ hygrothermal effectiveness or the effect of different weather conditions on their hygroscopic properties would be extremely beneficial. Both Building Energy Simulation (BES) and Computational Fluid Dynamics (CFD) are being used in the construction sector to determine energy balance requirements. It is possible to conduct long-term unsteady analysis using the BES methodology because the profile of physicochemical characteristics is consistent all across a given region, enabling this. When compared to the BES methodology, the CFD methodology involves breaking down the overall building volume into smaller elements or volumes through the usages of numerical meshing. These findings lead to a significant increase in computing power during unsteady state simulation studies. As a result, it is critical that the construction industry takes substantial steps to developing models as well as methodologies that can be used to forecast potential improvements. Building simulation tools must integrate efficient computing techniques while taking into account unique aspects, such as the coupled heat, as well as the moisture transfer through all of the envelope. The wide range of computational methods used by scientists and engineers depends on the application [20,21,22,23,24].

Improving and evaluating energy performance necessitates the use of a precise hygrothermal model. One can distinguish between white-box models, which are based on a physical understanding of the system, and energy balance equations. Black-box models, including the COMSOL Multiphysics and Transient System Simulation (TRNSYS) programs, which use only measured input/output data, as well as statistical estimation methods, including Artificial Neural Networks (ANN), without physics-dependent models, have been commonly used to obtain these results. Each method has pros and cons of its own. M. H. Benzaama and co-researchers [25] conducted a comparative study of these models. Hearing loss, sleep disorders, fatigue, heart disease, and other physiological and psychological issues are just a few of the health effects that are now widely recognized as being associated with excessive noise in the workplace and in daily life [26,27]. As a result, it is critical to keep household noise levels under control. Noise pollution can be minimized by using fibers’ sound absorption substances. Acoustic comfort (for example, speech intelligibility) could also be improved by using sound absorption materials to control reverberation time in concert halls, exhibition halls, workplaces, opera houses, and some others [28,29].

The construction industry relies heavily on fibrous material with a dual insulating material (sound along with thermal) [30,31]. As a result of their high specific surface area, elevated acoustical performance, and affordable price, some traditional fibrous insulators such as glass fiber and mineral wool are commonly used in sound absorption applications. A total of 60% of Europe’s market for insulating materials in 2005 was made up of glass fiber and mineral wool. In addition, organic foamy substances (such as polystyrene and polyurethane) account for 27% of the market and other materials account for 13%. Although the acoustic and thermal insulation properties of glass fiber and mineral wool are significantly superior, it could be neglected that breathing fibers and particles could indeed cause irritation as well as lay-down within lung alveoli, which can cause a few potential health problems [32,33,34,35]. Natural fibers, however, have a lower influence on the atmosphere than synthetic substances. When it comes to synthetic fibers, high-temperature manufacturing processes, as well as petrochemical sources, are often used to produce synthetic fibers, resulting in a substantial carbon footprint [36]. According to a Life Cycle Assessment (LCA), synthetic materials use more energy and have higher global warming possibilities from cradle to platform installation. Since conventional sound absorbers are harmful to the ecosystem, it is important to look into ecologically friendly alternatives. The low toxicity of natural fibers and the fact that they are safe for humans make them ideal substitutes for traditional sound absorbers. Because of their biodegradability, outstanding sustainability, abundance, and environmentally friendly nature, natural fibers have been referred to as green materials. Not to mention they have a substantially lower carbon footprint than synthetic equivalents [37,38,39,40], which is another advantage of organic fibrous acoustic absorbers. According to research, mineral wool is considered to be a natural fiber [41,42,43]. With all the above facts and figures in mind, the current review focuses on the hygrothermal and acoustic behavior of natural-fiber-reinforced polymer composites. Animal and vegetable fibers make up the bulk of the natural fibers considered in this study. Hybridization of fibers with synthetic fibers was also carried out in many studies. Such consolidation has also been made along with the synthesis and preparation of hybrid fiber-reinforced polymer composites.

## 2. Hygrothermal Behavior of Polymer Composites

### 2.1. Carbon Nanofibers Hybridized with Natural Fibers

Flax fibers are considered to be the most hydrophobic fibers among various natural fibers. The consequences of carbon nanofibers’ (CNFs) composition on flax-fiber-reinforced epoxy (FFRE) thermoplastic composite hygrothermal aging behaviors and mechanisms were examined in many of the previous studies. It took 180 days to test CNF/FFRE laminates comprising 0.25–2.0 wt.% CNFs. The properties of water absorption and tensile strength, as well as thermodynamics, have all been examined. According to the findings of the study, CNFs had a significant impact on the FFRE laminates’ hygrothermal properties. Due to the water obstacle characteristics of CNFs with FFRE laminates, water uptake was considerably lowered. Due to increased matrix stability, as well as improved interface bonding, CNFs/FFRE laminates had better tensile and thermodynamic characteristics. FFRE laminates’ hygrothermal durability can be enhanced by having the appropriate balance of CNFs, as shown in this analysis [44].

FFRE laminates were reinforced with bi-directional flax fiber fabrics. 240 g/m^2^ area density, as well as a standardized thickness of 0.16 mm, were measured for flax fabrics in this study. For the binder of the FFRE laminates, an epoxy resin containing the main agent as well as the curing agent was used. In the FFRE laminates, CNFs were used as nanofillers. CNFs had an average size of 100 nm in diameter and 50–200 m in length. A graphic depiction of the manufacturing process of CNF-altered FFRE composites is demonstrated and also discussed in detail. For 30 min, acetone was mixed with four different concentrations of CNFs (that is, 0.25%, 0.50%, 1.0%, and 2.0% by mass of said epoxy resin) that were dehydrated inside an oven to eliminate whatever moisture they had before being weighed. Second, the CNFs with the acetone mixture were sonicated for 6 h at 60 °C before being placed inside an ultrasonic cleaning solution for scattering. For uniform CNFs with epoxy dispersion, the acetone/CNFS mixture was added to the epoxy primary agent and agitated mechanically before ultrasonic dispersion. Mechanical stirring entirely volatilized the mixture’s acetone [45,46].

**Figure 2:**
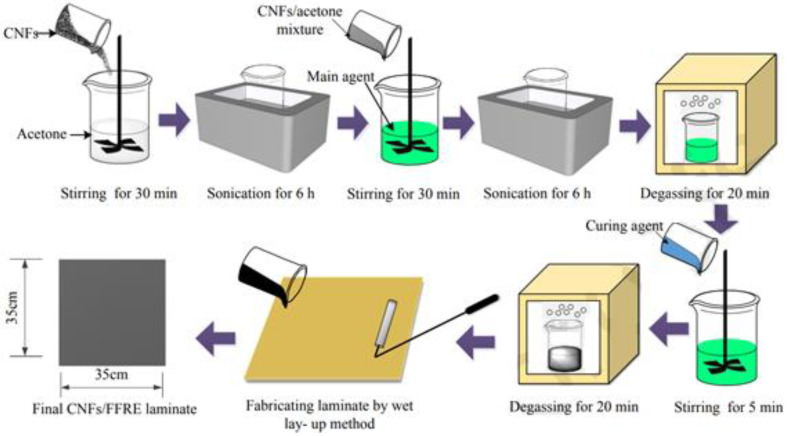
Fabrication of flax carbon nanofibers polymer composite (Adapted from the reference [42]).

The CNFs with the epoxy mixture were kept inside a drying oven for around 20 min to eliminate the bubbles. The curing agent was incorporated into the CNF/epoxy combination and stirred for 5 min at a low speed (a rotational speed of 500 rpm). For the next 20 min, the mixture was held in a vacuum oven to remove any residual gas. Wet lay-up was used to make the CNFs with FFRE laminates of two plies of bi-directional flax fiber fabrics with 350 mm × 350 mm measurements. Fabrics made from flax fibers were woven in the same direction of laying. CNFs with FFRE laminates had first been dried for 24 h at room temperature, then for another 24 h at 60 °C in the oven. In order to age as well as test the CNFs/FFRE laminates, they were kept in distilled water at various temperatures for 168 h (7 days). In the curing process, CNFs with FFRE laminates measured 0.2 to 1.3 mm in thickness. About 0.25 seemed to be the fiber volume ratio of said laminates. The epoxy binder to flax fabric laminate mass ratio was approximately 9:4. F1.0, F0.25, F0, F0.5, and F2.0 were used to indicate the FFRE as well as CNFs with FFRE laminates comprising 0.25%, 0.50%, 1.0%, and 2.0% of CNFs, respectively [42,47,48]. The laminates were cut into samples for every category of experiment in this analysis. Four types of samples for the water absorption, dynamic mechanical analysis (DMA), tensile, and FTIR experiments are displayed in Figure 2.

The hygrothermal aging behavior of CNF/FFRE composite laminates since adding CNFs throughout distilled water were explored, as well as the aging mechanism itself. The following inferences can be derived from this research: The hygrothermal aging behavior of FFRE laminates was improved by the addition of CNFs. Of the CNF/FFRE laminates tested in this analysis, those containing 1.0 wt.% CNFs had the best hygrothermal aging behavior. However, even with a 2.0 wt.% rise in the quantity of CNFs used, the absorption of water of the CNF/FFRE composites remained less than with the genuine FFRE laminates (1.0 wt.% CNFs). As a result of the hydrophobic CNFs filling in the matrix and creating a tortuous diffusion way for water molecules, the FFPR laminates were more resistant to water penetration. Tensile properties, as well as the elastic modulus of CNF with FFRE laminated under hygrothermal conditioning, improved as the CNF content rose to 1.0 wt.%.

As a result of this, SEM images of fractured samples showed that the addition of CNFs to the epoxy matrix not only increased epoxy matrix stability but also strengthened interface bonding between flax fibers and the epoxy matrix. The mechanical properties of CNFs with FFRE laminates before as well as after hygrothermal aging were higher than that of genuine FFRE laminates. Since CNFs made an obstruction within the epoxy matrix of FFE laminates, the cross-linking density of the epoxy matrix increased. Due to the hydrogen bonds formed by type II bound water, the glass transition temperature (Tg) of CNFs with FFRE, as well as genuine FFRE, significantly increased after immersion in water. There were slower aging rates for CNFs with FFRE laminates subjected to a hygrothermal environment, as evidenced by changes in relative bands as well as functional groups in the FTIR spectrum. Due to CNFs ability to strengthen the epoxy matrix as well as inhibit water diffusion in laminates, one such result was achieved. In this analysis, CNFs at a concentration of 1.0 wt.% had the greatest impact on trying to slow the aging of FFRE laminates. Consequently, FFRE laminates can be improved in their long-term hygrothermal durability by incorporating an adequate quantity of CNFs into laminates [49,50].

### 2.2. Palm and Hemp Fibers

Though fully biodegradable natural fibers are hydrophilic in nature, their hydrophobicity was improved by the addition of nanofillers in natural-fiber-reinforced polymer composites. Natural-fiber-reinforced mortars were tested experimentally for hygrothermal and mechanical properties in many studies. The percentages of fibers derived from palm stems (PS) in nano scale, as well as hemp, (HF) were compared. Using a scanning electron microscope (SEM), we discovered that the PS fibers had rough surfaces and complex microstructures. The fibers were treated to reduce their hydrophilicity before being incorporated into the mortar. Both PS and HF fibers had their water absorption reduced significantly thanks to the treatments used. Low thermal conductivity, as well as excellent moisture buffering, were also found in the mortar mixtures containing these fibers. The investigated mixtures had moisture buffer values (MBVs) ranging from 2.7 g/% HR·m^2^ to 3.1 g/% HR·m^2^, indicating their good moisture regulator properties. When the fiber mortar mixtures were aged for 28 days, the expected outcomes emerged: extremely high porosity as well as low compressive strength (between 0.6 and 0.9 MPa). Materials developed for thermal insulation and construction filling in this analysis had low environmental footprints [51].

The results of the experiments described in this paper led to the development of a new natural fiber. After successfully extracting and characterizing the fiber from the date palm, it was incorporated into mortar mixtures at varying dosages in various ways. The effect of these fibers on the hydrothermal characteristics of mortar was studied. Lignin, hemicellulose, and pectin deposit, were visible on the surface of the PS fibers. When examined under a microscope, it was discovered that the PS fibers have a morphology comparable to coir fibers, as well as a microstructure composed of an arrangement of elementary fibers that had decided to open on their surfaces. In addition, the new PS fibers are hygroscopic and have a high capillary condensation. Up to 50% of their water absorption was diminished as a result of the addition of the fibers’, and implantation in hydrophobic resin also decreased their hydrophilic properties. In comparison, 185 mW per (m·K) was the thermal conductivity of a control mix in the dry mortar mixes containing 5% PS and HF fibers. To put this in perspective, a 245% and 200% increase over the control mortar was seen in the wet mixture. In addition to the percentage and orientation of fibers in the matrix, water content has an impact on the heat conductivity of such a mixture. Regardless of the fiber content, the fiber mortar blends demonstrated good porosity as well as water absorption compared to the control mixture. Porosity, as well as water absorption, increased as a result of fiber content. Improved moisture buffer capacity, as well as lower thermal conductivity and compressive strength, were achieved as a result of these changes. Regardless of fiber content, all of the investigated fiber mortar mixtures had an MBV exceeding 2 g/% HR·m^2^. The better moisture absorption behavior was observed with 5% of natural fibers [52,53].

The creep/recovery behavior of three high-grade composites manufactured of green epoxy, flax, and hemp fibers were investigated in the literature. The stress level and hygrothermal conditions were examined. In this study, it was found that as load and environmental severity increased, so did the levels of extremely rapid, time-delayed and residual strains. Material time-delayed tensile strains seem to be higher during creeping than during recovery. Moreover, a stiffness effect is observed as the body begins to heal, and the stiffening effect is observed. Using the recovery function, an anisotropic viscoelastic existing legislation can identify the material’s viscoelastic properties after creep. Stress levels and environmental conditions do not affect the relaxation time function. For example, the viscosity parameter rises dramatically with increasing stress levels and harsh environmental conditions. Stiffening and irreversible mechanisms, rather than viscoelastic behavior, are to blame for the recovery behavior’s stress-level dependence [54].

### 2.3. Wood Fiber

**Figure 3:**
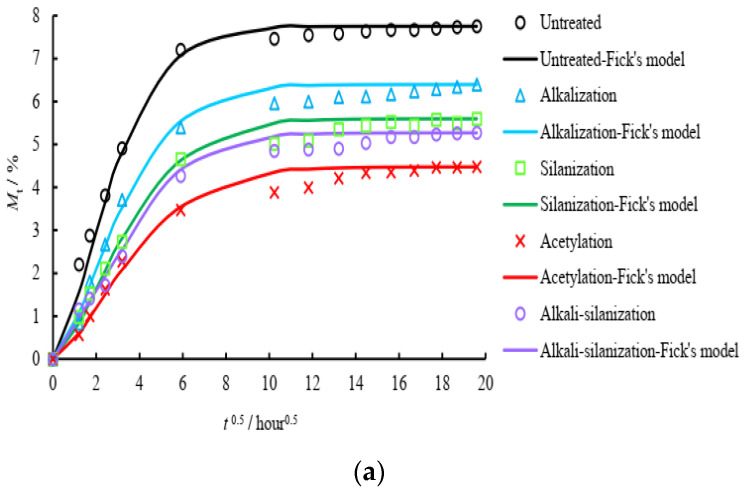

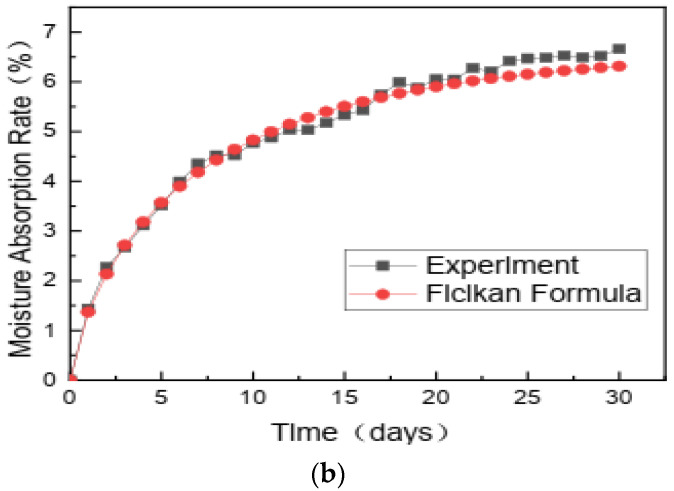
(**a**,**b**) Hygrothermal aging curves of polymer composites (Adapted from the references [49,55]).

There are a slew of new insulation materials currently being designed, tested, and refined in order to enhance the energy efficiency of both newly constructed and existing structures. To save energy as well as reduce environmental impact, bio-sourced materials have emerged as one of the new insulation options available today. In recent years, wood fiber has become one of the most popular natural insulation materials. There are numerous benefits to using this type of insulation, including its ability to store moisture, which improves indoor air quality, as well as its natural tendency to accumulate and mitigate noise. Few studies investigated hygrothermal modeling and the performance of wood nanofiber insulation in constructing implementations in order to assess its efficiency. A mathematical model was used to describe heat as well as mass transfer within wood fibers as porous media using numerical methods. Experimental data, thermal conductivity, heat capacity, and sorption/desorption isotherms of the wood fiber material will be first determined for this purpose. In order to verify the accuracy of the numerical model, an iterative procedure is used in both controlled as well as uncontrolled environments. For this experiment, a wood fiber sample measuring 50 cm by 50 cm and measuring 8 cm thick was used. Samples at x = 2 cm and x = 4 cm show very good agreement between the measured and modeled temperatures as well as relative humidity evolutions, with mean differences between the two of 0, 21 °C at 4 cm and 1 °C at 2 cm. The measured maximum distinctions again for relative humidity seem to be: 5.5% at x = 2 cm but also 4.5% at 4 cm, respectively. This is consistent with the results of the model. Predicting wood fiber insulation’s heat transfer as well as hygroscopic behavior will help researchers learn more about the effectiveness of natural insulation substances [55,56]. Figure 3a,b denotes the moisture absorption rate curve for different polymer composites based on the traditional method of measurement and Fick’s model.

The hygrothermal properties of wood fiber insulation was studied both experimentally and numerically in this study. The heat and mass transfer in the porous media model developed by a few researchers [57] were used for the above bio-based material with a complex structure. This material’s response to microclimatic conditions as well as its hygroscopic map were first determined using a water vapor sorption curve. Experimentation was used to determine the material’s thermophysical and hygroscopic characteristics in simulating the material’s behavior under real-world conditions. Thermal conductivity increased between 0.045 and 0.06 W per m1.K1 for an increase in water content from 2 to 18%. Correspondingly, for almost the same moisture content range, volumetric heat capacity seemed to have an increasing shape ranging between 250,000 and 350,000 J/m^3^ K. Using thermocouples and humidity sensors, we experimented with various environmental conditions on a representative wood fiber sample.

A numerical model was developed and tested against experimental data from controlled and uncontrolled ambiances using boundary conditions of air temperature as well as relative humidity there at the interaction. First, the temperature was kept constant, while the relative humidity was kept constant. Then, a transient temperature was applied, while the relative humidity was kept constant. The third scenario studied a coupling of temperature and humidity variations in the air. Validation of wood fiber behavior under controlled conditions was confirmed by comparing calculated and observed temperatures and relative humidity there in three configurations. Due to an inability to forecast the hygrothermal behavior of the wood fibers in real ambient conditions, the sample was tested under uncontrolled conditions. The temperature and relative humidity changes in the sample were in excellent agreement, with an upper limit difference of less than 5% for the relative humidity as well as a maximum difference of 0.21 °C again for temperature distributions. With these findings, we can verify the mathematical model for the stochastic heat and mass transfer within wood fiber insulation in a real-world setting, highlighting the effect of coupling within bio-based materials. More accurate predictions of the hygrothermal behavior of wood fibers can be made using numerical and experimental approaches. To understand better the wood’s nature, additional characterizations, such as water vapor permeability persistence or porosity analysis, can be performed. Previous studies predicted the use of hysteresis models to describe the isothermal behavior of sorption as well as desorption [58,59].

### 2.4. Flax-Fiber-Based Composites

Due to their own renewable green origin as well as high tensile strength, flax fibers are becoming a popular research topic. Because of their water absorption as well as poor adhesion to the polymer matrix, composites made from natural fibers have low interfacial strength. For better flax/polypropylene composite performance, this study uses a hybrid chemical therapeutic technique that combines alkali (sodium hydroxide) and silane treatments. SEM, FTIR, AFM, XRD, and a microfiber tester were used to examine the surface morphology, microstructure, chemical composition, wettability, crystallinity, and tensile properties of a single flax fiber before and after chemical treatments. Due to alkalization, hemicellulose, and lignin being removed from the fiber surface, there was a reduction in moisture absorption in the composites. The polypropylene matrix compatibility was improved after alkali-treated flax fibers were exposed to silane treatment, and the composites’ moisture absorption was further reduced following alkali–silane hybrid chemical treatment. At about the same time, the strength of the interfacial bond between flax and polypropylene was significantly improved. It is clear from these findings that the hybrid chemical therapeutic approach for flax with polypropylene composite materials has a lot to offer the plant fiber composite company, particularly in terms of expanding the use of chemical treatment methodologies in the field [60,61].

**Figure 4:**
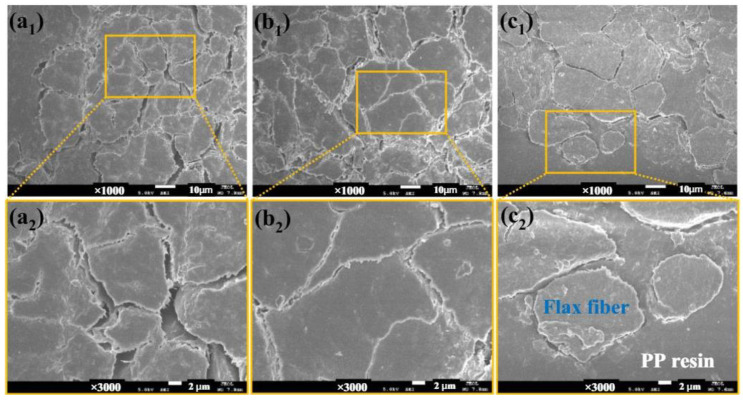
Morphology of flax/PP composites: (**a_1_**,**a_2_**). untreated flax/PP composites, (**b_1_**,**b_2_**). alkali-treated flax/PP composites, and (**c_1_**,**c_2_**). alkali silane-treated flax/PP composites (Adapted from the reference [48]).

Due to the other environmental and economic advantages, organic FRPs (fiber-reinforced polymers) are being targeted in a variety of industries. One of the most common natural FRPs is FFRPs (flax FRPs). A key factor in the FFRP’s practical engineering application is its long-term durability, as well as its performance. FFRP’s creep, as well as its dynamic mechanical properties, were investigated experimentally. Cranking up its creep as well as dynamic mechanical performance saw less progress. An investigation into the effects of surface treatment on the creep as well as the dynamic mechanical behavior of FFRP was conducted in this article under hygrothermal aging conditions. These findings demonstrated enhanced creep, dynamic effectiveness, and reduced moisture content in the FFRP after surface treatment. For the substances studied, a fractional-order creep framework was developed. The analysis indicates that fractional calculus seems to be an effective tool for characterizing FFRP’s creep behavior accurately and precisely with fewer features than the conventional creep model does [62]. Figure 4 shows the surface morphology of flax-fiber-reinforced polypropylene composites, before being subjected to hygrothermal aging. Figure 4(a_1_,a_2_) represents the untreated composites, Figure 4(b_1_,b_2_) represents the alkali-treated composites, and Figure 4(c_1_,c_2_) represents the alkali silane-treated composites.

### 2.5. Mycelium-Based Composites

In order to better understand mycelium-based composites (MBCs), this study will evaluate their effectiveness as a foam-like wall insulation. The composite’s performance has been maximized by using a variety of substrates. A longer growing period resulted in a denser outer layer of mycelium in MBC, which improved water resistance owing to mycelium’s hydrophobicity. More than any other parameter of MBC, substrate choice has the greatest impact on thermal conductivity, as well as mechanical characteristics. The effects of aging and MBC’s moisture buffer capacity were also examined in this study. The accelerated aging test (that is, drying and wetting cycles) showed that MBC not only preserved its functional performance but also constituted excellent moisture buffering capacity. To inactively monitor and control indoor relative humidity as well as thermal comfort, MBC can be used as an internal wall insulation layer, which is a layer in vapor-permeable built environment systems [63].

### 2.6. Coconut Fiber

As an “exotic” insulator, coconut fiber insulators clash with the skepticism of their thermohygrometric behavior, especially in the context of covering technology including green roofs, which are a workable alternative often implemented in the case of green solutions or nearly zero energy structures. Green roofs are a viable option for both new and existing buildings because of their high thermal performance. As an alternative to synthetic insulators, coconut fiberboards (CF) were used to study the thermohygrometric behavior of concrete (CLS) and cross-laminated wood (CLT), respectively, on ten different green roof scenarios. In the end, the results indicate that coconut fiber insulations seem to be significant compared to organic and inorganic materials, and that the doubts about their applications, including green roofs, are connected to engineering solutions for application in the market and their own diffusion between both the building materials, rather than their own hygrothermal characteristics [64].

### 2.7. Jute-Fiber-Based Composites

Reinforced composites made using VARI technology were evaluated for changes in hygroscopic properties and the impact of hygrothermal aging on their mechanical properties. The findings demonstrate that the composites’ first-stage moisture absorption follows Fickian diffusion closely. The Fickian equation’s diffusion coefficient-temperature correlation was corrected using experimental data. This suggests that the correction was successful, given the small discrepancy between the experimentally based moisture uptake curve and the one anticipated by the altered equation. Temperature also has an effect on the composite strength because the resin-dissolving process becomes more intense. At stage one of this analysis, the hygroscopic conduct of jute fabric composite materials corresponded well to Fickian diffusion. At 40 °C, there was some minor deviation between the modified equation’s hygroscopic curve and the experimental-data-based curve, which proves that the correction is effective, as well as its possible utilization to predict other temperature-dependent hygroscopic curves of jute fabric composites. As the temperatures rise, the resin appears to dissolve, resulting in degradation of the composites’ tensile properties, as shown by the crucial tensile loads measured at 25 °C, 40 °C, 55 °C, and 70 °C, which were 56.42 MPa, 52.17 MPa, 33.51 MPa, and 16.60 MPa, respectively [65].

Polylactide (PLA) composite materials were tested for their aging characteristics in the hygrothermal environment. To create the material, the film-layering hot-pressed technique was used as a fabrication process. Saturated vapor at 70 °C was used to age both uncoated and adhesive-tape-coated samples. While the samples were aged, the rate at which they absorbed moisture was charted. There were three stages of moisture absorption in uncoated samples: a stage of short and rapid moisture uptaking; a stage of slow, steady uptaking; and a stage of an abrupt, extremely quick uptaking. Different stages of the aging of the samples were observed in their microstructures. Pores, microcracks, delamination, and complete structural relaxation were among the most common aging-related defects. The coating appears to be an effective way to slow down the aging and moisture absorption processes. The gel permission chromatography (GPC) results indicated that the PLA matrix had been severely degraded in a hygrothermal atmosphere. After aging, tensile strength reduced significantly [66].

### 2.8. Rice-Husk-Based Composites

For in-house development, NFPCs (natural fiber plastic composites) are most commonly used as deck boards. The moisture adsorption or desorption behavior and hygrothermal dimensional stability of these composite materials with high fiber content as well as hollow profiles are put into question when exposed to a wide range of climate conditions. Commercial decking panels made of rice hull and high-density polyethylene (HDPE) were tested under simulated adverse climate environmental conditions in this analysis. When exposed to 93% RH and 40 °C for 2000 h, the specimens gained 4.5% of their original weight in water content. A significant (7.1%) swelling of the samples’ walls occurred at the same time, which resulted in a longitudinal bowing of about 5 mm (based on 61 cm longboards). After another 2000 h of exposure to 20% RH and 40 °C, both expansions, as well as bowing, partially managed to recover. When exposed to a variety of weathering conditions, the samples took longer to reach a new humidity balance than a new dimensional equilibrium. Deformation, including swelling and bowing, was largely due to the presence of moisture. Additionally, temperature affected the rate and amount of moisture adsorption, as well as causing straightforward thermal expansion or contraction [67,68].

### 2.9. Agave-Fiber-Based Composites

The longevity of agave organic fiber-reinforced polymer composite materials was tested under hygrothermal aging conditions using three distinct pre-treatment methods: alkali hornification, liquid hornification, and alkali treatment. Moisture diffusion analysis was performed on the composite specimens using standard test procedures. Distinct hygrothermal aging conditions were applied to the standard test samples taken to examine the effects of different treatment conditions. Alkali hornification contributed to a 27% decrease in water preservation in the agave fibers after four consecutive hornification cycles. Water hornification reduced water retention by only 6% in similar circumstances. In direct contact with liquid, the alkali-hornified composite materials gained 4.1%, 4.3%, and 4.7% mass gain, respectively, between 25 °C and 75 °C. At the same temperatures, alkali-treated composites gained mass at rates of 4.3%, 4.5%, and 5.1%, while water hornification gained mass at rates of 5.5%, 5.9%, and 6.1%. There were mass gains ranging from 0.9% to 1.3% for the alkali-hornified composites when humidity levels were kept constant between 25 °C and 75 °C. Comparable temperatures led to mass gains of 0.6%, 1.05%, and 1.2% for alkali-treated composites, as well as 0.66%, 1.07%, and 1.3% for water-hornified composites. Fiber pre-treatment methods have an impact on the moisture resistance of agave natural fiber composites, as shown in the current study. Microstructural investigations bolster the findings [69].

### 2.10. Silk- and Ramie-Fiber-Based Composites

Bio-composites reinforced with silk or henequen natural fiber were evaluated for their hygrothermal properties in the literature. Compression molding was used to create the bio-composites. For 1000 h, the bio-composites were kept at 60 °C and 85% RH. Deterioration of the bio-composites’ achievement was primarily due to PBS matrix degradation, and the bio-composites deteriorated more slowly than the PBS matrix. For the bio-composites exposed to 60 °C and 85% RH for 1000 h, the storage modulus of the fibers decreased by 20% and 50%, correspondingly, when compared to the initial specimens [70]. Short ramie fibers and poly (lactic acid) (PLA) composites were prepared by a combination of extrusion and injection molding. For the ramie with PLA composites at 60 °C, water absorption and aging were studied. In this study, the water absorption and mechanical properties of something like the ramie with PLA composite materials with immersion time were revealed and evaluated by comparing them to those of PLA alone. Differential scanning calorimetry along with gel permeation chromatography was used to measure the crystallinity and molecular weight of neat PLA as well as of ramie with PLA composite materials. The water absorption and mechanical characteristics of the short ramie fiber were reported to be influenced by the results. As a result of the hydrophilic nature of short ramie natural fibers, composites of ramie with PLA showed greater saturation weight gains and diffusion coefficients than did neat PLA alone. Age-related deterioration in tensile and flexural strength was extreme. Furthermore, PLA degradation was found to be amplified by ramie in a hygrothermal environment. Scanning electronic microscopy was used to examine the fracture surface morphologies of clean PLA as well as of ramie with PLA composites, with varying immersion times. PLA degradation and ramie with PLA bond de-bonding led to the microcracks and voids [71,72].

### 2.11. Luffa Cylindrica Fiber-Based Composites

The mechanical and hygrothermal properties of polyester with luffa composites were examined in relation to luffa fiber surface chemical modification. The matrix was made up of unsaturated polyester resin. Luffa fibers that were untreated, alkali-treated, combined-processed, and acetylated were used. The fibers of the luffa plant were studied using scanning electron microscopy as well as infrared spectroscopy. Tests for the composites’ mechanical characteristics were conducted using a three-point bend test. Saturation in filtered water at 25 °C was used to test the fibers and composite materials for water absorption. Improved mechanical characteristics were achieved through acetylation treatment. The infrared assessment showed that the procedure reduced the hydrophilic behavior of the luffa fibers, which improved their bonding to the polyester matrix. The diffusion coefficient and maximum water absorption of luffa fibers were both reduced as a result of the chemical modifications made to their surfaces. For the fibers tested in this analysis, the diffusion methodology was “Fickian” at the beginning of immersion but became more complicated at the end. Composite materials showed similar results when immersed in water at previous phases. Composite materials exposed to external loads were found to affect diffusion. Water absorbed by the body increases in volume at an even faster rate as the load is an excessive amount [73]. Table 1 consolidates the various studies carried out on different natural-fiber-reinforced polymer composites along with the test conditions and moisture absorption values.

## 3. Sound Absorption Behavior of Polymer Composites

**Figure 5:**
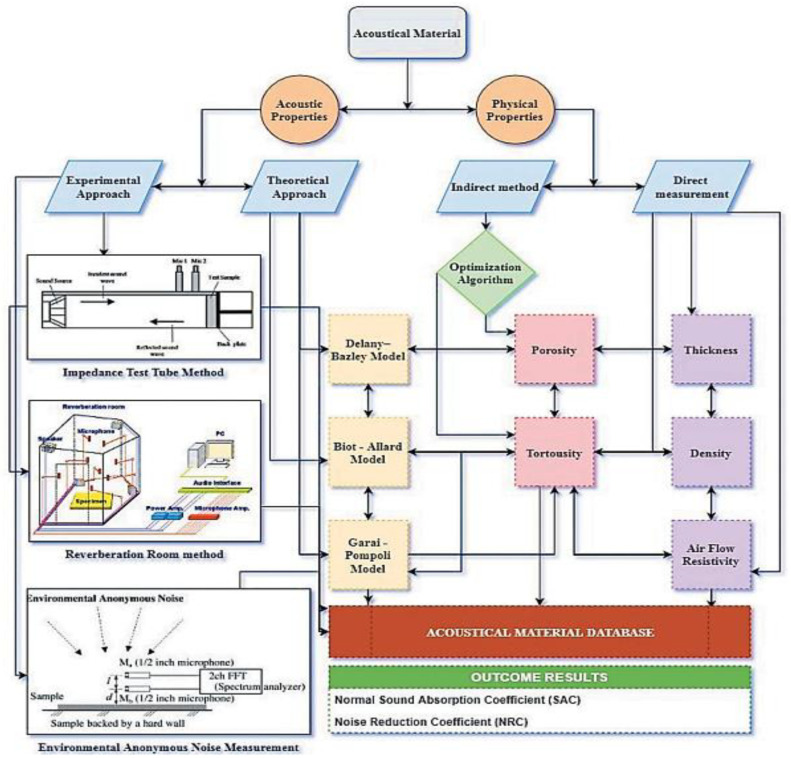
Methods of measuring sound absorption properties (Adapted from the reference [104]).

Nowadays, humans are at risk of developing serious diseases as a result of increased noise pollution caused by external factors. A filler or panel made from bio-based materials can help reduce undesirable noise in workplaces and also homes. An acoustic absorber made from eco waste fibers (remains upon harvesting) has been developed and tested. The gleaning methodology, which is the method of acquiring field leftovers, is used to gather the eco waste fibers [104,105,106]. Figure 5 shows the methodology of measuring sound absorption along with the equipment and different mathematical models. The following sections deal with the determination of the sound absorption coefficient (SAC) of various natural-fiber-reinforced polymer composites and the possibility of using such composites in acoustic insulation applications.

### 3.1. Yucca-Gloriosa-Based Composites

Natural fabrics, with their numerous environmental, physical, mechanical, and sound-absorbing benefits, have revolutionized the manufacturing of organic fibers. Investigators are paying expanding consideration to the acoustic behavior analysis of natural fiber composites, known as “The Green Fibers,” because of the additional funding they can generate by absorbing sound. Using a mathematical imitation as well as an optimization approach, this analysis aims to optimize and replicate the sound absorption behavior of Yucca Gloriosa (YG) composites. The alkaline treatment of the fibers was used in this investigational cross-sectional study to manufacture the natural acoustic composites. In order to enhance sound absorption, Response Surface Methodology encouraged the design of experiments as well as the determination of the optimal quantity of alkaline treatment specifications (NaOH concentration as well as immersion time) (RSM). Additionally, an impedance tube structure was used to determine the YG fiber’s sound absorption coefficient (SAC) (ISO10534-2 standard). Delany–Bazley (DB) and Miki analytical models were tested in MATLAB software to see if they could be applied to predicting the SAC of natural composites. At all frequencies, a comparison of the procured SAC values showed that the optimized composites had higher values than the untreated ones. There was an 18.92% increase in the Sound Absorption Average (SAA) Index, especially when compared to the raw composites. Moreover, the empirical models, as well as the experimental data in the low and mid-range of the one-third octave band, were found to be in good agreement. Optimum alkaline treatment and empirical-model-based SAC forecasting are deemed appropriate strategies for acoustic implementations because of the prominent advantages of natural materials and their widespread use [107].

### 3.2. Oil Palm Trunk

Because they are more sustainable and can be replenished, natural fibers are increasingly being used to replace synthetics in sound-absorbing materials. Research on oil palm trunk (OPT) fiber’s SAC as an acoustic material is presented here. All OPT specimens were examined for SAC using the impedance tube method (ITM). Including an average density of around 100 kg per m^3^, OPT’s natural fiber was used to create three different thicknesses of panels: 8 mm, 12 mm, and 16 mm. OPT’s acoustic efficiency was evaluated to be very good, with all samples almost reaching unity (0.9) at a high rate above 3000 Hz. The results also show that SAC varies between 0.5–0.85 at low frequencies below 500 Hz. 0.99 at a frequency range of 3000 to 6000, and 6400 Hz was found in the thickest natural fiber panel of 12 mm, making oil palm trunk an extremely promising natural fiber for use as a sound-absorbing content. Fiberglass, despite its excellent acoustical absorption properties, is not a good choice. Fiberglass has been linked to a number of serious health issues, including skin irritation and redness; eye, nose, and throat irritation; and even cancer. Fiberglass dust can cause bronchitis, difficulty in breathing, coughing, and perhaps even lung disease if it is inhaled too much. Only the acoustic properties, including the use of OPT natural fibers as a replacement for fiberglass, were examined in this study [108].

Additionally, natural fibers play an essential part in the design of ergonomic products. They reduce both the noise level as well as the health risks while also preserving a pleasant working environment. Using a frequency range of 0 to 6400 Hz, OPT fiber exhibits excellent sound absorption properties. SAC (alpha) = 0.99 was reached by some OPT fiber samples in this manner. OPT fiber was able to absorb 99% of the incident sound, while only 1% of the sound was reflected. The influence of OPT fiber thickness was discovered through sample characterization. The results clearly show that increasing the thickness of a material significantly reduces its absorption rate. Porosity decreases with increasing sample thickness because more fiber is present, reducing the sample’s porosity. In order for the OPT fiber to perform at a high SAC (alpha) when the frequency raises, the porosity of the fiber must lessen. Thus, the ideal OPT fiber thickness was found to be 12 mm. OPT fiber’s ideal density and thickness were the primary focus of this investigation. Because of this, a fiber with a density of around 100 kg per m^3^ was found to be the best OPT fiber. The sample’s maximum SAC (alpha) value was 0.99 under these circumstances. When compared to synthetic-based commercial products, the OPT experimental trial findings reveal that it has excellent acoustic characteristics [109].

Oil palm timber is one of several solid wastes that can be used for non-structural purposes. Numerous researchers concentrated on the strength of their materials and indeed the binder they use. The use of oil palm timber as an insulation material has only been examined in a few research findings. In order to better understand oil palm timber binderless panels’ thermal and acoustic properties, this study was conducted. Various particle sizes and pressing times were used to make panels from oil palm timber. Binderless panel properties were affected by particle size, but pressing periods were not, according to the findings. The greater the particle size, the greater the resistance to heat and sound, but the lower the density, the greater the water resistance, as well as the lower the bending strength. Large granules also resulted in the lowest heat conductivity (0.050 W/mK) and, indeed, the highest SAC (0.33). Flexible strength and liquid absorption usually range from 4.21 to 8.18 MPa and 84.51%–119.06%, respectively. This study’s research results suggest that oil palm timber binderless boards can be used in acoustic insulation applications [110].

### 3.3. Sugarcane-Bagasse-Based Composites

Sugarcane bagasse fibers are considered to be the most porous of all fibers and can render better acoustic behavior when reinforced in composites. Few studies examined the thermal and acoustic properties of sugarcane bagasse and bamboo charcoal insulation specimens for use in the automotive industry. For thermal and sound insulation applications, sugarcane bagasse and bamboo charcoal fiber could be viable raw material sources. The primary use for natural fibers is sound absorption, and this is one of the most common applications. At this time, the natural fiber hybrid composite is more sought after by industry because of its advantages, such as low-cost, biodegradability, appropriate physical characteristics, etc. Bamboo charcoal and sugarcane bagasse fibers have been used to develop environmentally friendly sound-absorbing composite materials. Compression bonding was used to create five different types of natural fiber green composite from these fibers. An important factor in the widespread use of sound absorbers made from natural composites is their noise-control performance. The impedance tube technique was used to measure the SAC in accordance with ASTM E 1050. Using the ASTM standard, the physical characteristics of natural fiber composites were evaluated for all specimens, including the thermal conductivity, thickness, air permeability, porosity, and density. Natural fiber green composites absorb more than 70% of the sound resistance and provide the greatest acoustic absorption characteristics; these composite materials possess appropriate moisture resistance in wet environments without influencing the insulation or acoustic characteristics among these composites [111].

### 3.4. Kenaf with Waste Tea Leaf Fiber Composites

**Figure 6:**
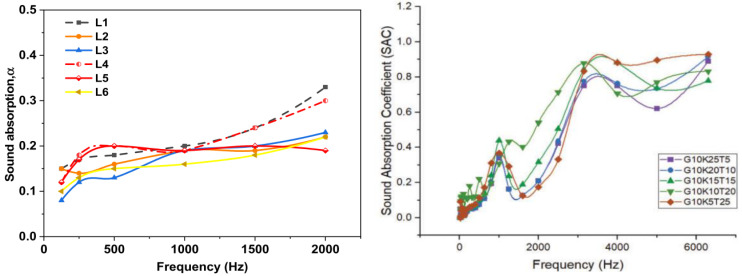
Sound absorption coefficients of polymer composites (Adapted from the references [110,112]).

Waste tea leaf fibers (WTLF) are most commonly used as a nanofiller in composites to increase the porosity of the composites. The purpose of this study is to examine the structural and sound absorption properties of manufacturing waste tea leaf fiber, kenaf, and E-glass fiber-reinforced combination epoxy composites. WTLF and kenaf fibers were first treated with sodium hydroxide at a concentration of 5%. Compression molding was used to create hybrid composites with a 40-to-60-to-1 fiber-to-matrix ratio. Mechanical and sound absorption tests were conducted on the manufactured hybrid composites in accordance with ASTM standards. The composites containing 25% kenaf and 5% WTLF had better mechanical properties, while the composites containing 25% WTLF and 5% kenaf had better sound absorption properties. Scanning electron microscopy was used to study the surface morphology of something like the shattered samples, such as fiber pullout and matrix crack. Research on alkali-treated hybrid composites showed that polymer and fiber interfacial bonds were much stronger than those in untreated composites [112]. Figure 6 denotes the sound absorption coefficient variation of various polymer composites. In Figure 6, L denotes oil palm timber particles of different particle sizes, G denotes glass, K denotes kenaf, and T denotes WTLF.

The acoustic properties of a natural fiber panel are determined by the fiber’s physical properties. In this study, a chemical treatment was used to enhance the sound absorption properties of kenaf fibers. Sodium hydroxide (NaOH) treatment from 1% to 8% concentration levels seems to be the goal. SEM was used to observe the effects of NaOH treatment on the kenaf fiber strands. Measurements were made using an impedance tube to determine the SAC and noise reduction coefficient (NRC) values, respectively. The diameter of kenaf fiber decreased as the NaOH concentration increased, according to the results. FTIR analysis proved that the strand diameter decreased as a result of the removal of the hemicellulose and the lignin layer of the strands. Treatment with 6%, 7%, and 8% NaOH resulted in elevated SAC at high frequencies (>2500 Hz) than untreated fiber. Specimens with NRC values of 0.67 were found. Thinner strands of kenaf absorber were found to improve sound absorption, while a 6% NaOH concentration was found to be optimal for treating kenaf fiber for sound absorption [113].

### 3.5. Sisal and Palm Fibers-Based Composites

Hybrid composite materials made of palm fiber and sisal fiber were examined in this study for tensile, flexural, impact, and sound absorption properties. A compression molding technique was used to create three distinct hybrid composites, each with a different weight ratio with 5%, 10%, and 15% of palm fibers. The tensile, flexural, and impact tests for the three hybrid composites were carried out in accordance with the appropriate ASTM standards. According to these results: The combination of epoxy resin (65%), cellulose (20%), sisal (15%), and palm fiber (15%) yielded the highest tensile strength, flexural strength (22%), and impact strength (36%). Images of fractography showed fiber to fracture, surface, and fiber pull out, as well as the deformations of tightly packed fiber and matrix stickiness. An impedance test on hybrid composites was used in accordance with ASTM E1050 standards to determine their sound absorption behavior. Due to the greater weight proportion of palm fiber (65% epoxy resin, 20% sisal fiber, and 15% palm) in the composite, it rendered a high SAC at frequencies of 1600 Hz, 2000 Hz, 2500 Hz, 3150 Hz, and 4000 Hz. Above that, the above hybrid composite exhibited a maximum SAC difference of 76.61% in comparison to the other three hybrid composites at a frequency of 4000 Hz [114].

### 3.6. Jute and Luffa Fiber-Based Composites

Before utilizing natural fiber-reinforced composite materials in different industrial applications, such as sound and vibration isolation, it is necessary to determine their acoustic characteristics. The acoustic effectiveness of jute and luffa fiber-reinforced biomaterials is examined in this study by varying the sample thickness as well as the fiber and resin ratio. The impedance tube method was used to measure the SACs and transmission losses (TLs) of jute and luffa composite samples of various thicknesses, fibers, and epoxy ratios. The thickness-dependent characteristics of the SAC/TL of jute and luffa composites were identified in the low-, medium-, and high-frequency ranges. The acoustic characteristics of jute and luffa composite materials as a function of frequency were ascertained by determining the fiber/epoxy ratio. In addition, mathematical techniques were used to estimate the SACs and TLs of various natural-fiber-based specimens with varying thicknesses, and with the conceptual and experimental findings were compared and evaluated [115].

### 3.7. Coconut Coir and Oil Palm Fruit Bunches, with Pineapple Leaf

Three different types of natural fiber composites were compared for their sound absorption properties. To make the fibers, oil palm waste and pineapple leaves were combined with coconut coir and pineapple leaf fiber. Sample thicknesses ranged from 10 to 20 mm, and two fiber density options were used to determine the SAC. For the evaluation, a non-sophisticated impedance tube with 2 microphones was utilized. The transfer function of the two microphones was well within the range of 200 Hz to 3000 Hz, and was used to determine the SAC. Three types of fibers were found to reduce reflected sound at a higher frequency than previously thought. The high point of an absorption coefficient is shifted lower in frequency as the density and thickness of such fibrous material raises. Moreover, the SAC of pineapple leaf fiber is the highest among several other fibers. Pineapple fiber absorbs sound energy due to its small and uniform fiber diameter. Because of this, the SAC of coconut coir, as well as palm hollow fruit bunches fiber, is likely to be lower, particularly within the frequency range of measurement [116].

The composite material microperforated panel (MPP) created from coconut fiber and polylactic acid (PLA) bio-composite material was for the bio-composite microperforated panel (BMPP). PLA served as a matrix for the BMPP specimens made from coconut fiber. The SA performance of the BMPP specimen was determined using an impedance tube technique, whereas the porosity of the specimen was determined using a porosity tester. The SA performance of BMPP and steel MPP was compared. The BMPP with different percentages of coconut fiber and PLA had different sound absorption performances because of the presence of pores and tortuous structures in the specimen. BMPP specimens were subjected to a scanning electron microscope (SEM) evaluation to better understand their structure [96]. Using both experimental and theoretical methods, this study examines the sound absorption properties of coconut coir fiber. Sound absorption coefficients were measured using samples of coconut coir fiber with different thicknesses and densities. ISO 10534-2 and ASTM E1050-98 guidelines were used to set up an impedance tube to measure the SAC. Thickness and density were studied using the Delany–Bazley model. This paper presents and compares the results of theoretical and experimental studies on SAC. When compared with experimental results, the Delany–Bazley model appeared promising, but further deviations were found. It was found that a 35 mm thick sample with a density of 220 kg/m^3^ had a SAC of 0.84 (2900 Hz). The transfer function technique of the impedance tube was used to estimate sound transmission loss (STL) for samples with 220 kg/m^3^ density (21 mm, 28 mm, 35 mm). Coconut coir fiber samples were found to effectively dissipate sound energy, resulting in acceptable sound absorption properties [117].

### 3.8. Finger Millet Straw and Darbha, with Ripe Bulrush Fibers

Few experimental trials focused on testing the sound absorption capabilities of natural fibers extracted from Eleusine coracana, Desmostachya bipinnata (Darbha), and Typha domingensis, or hybrid configurations There are a number of variables that influence the material’s ability to absorb sound, including its density, porosity, thickness, flow resistance, and tortuosity. For individual fibers or hybrid combinations, the length and type of the fibers play important roles. The hybridized fiber configuration was tested to see if stacking had an effect on sound absorption. All pairings had a lower SAC (alpha) in the 1000 Hz to 2500 Hz frequency range. For a 50 mm thickness, the darbha fiber had the best NRC of 0.86, whereas the ripe bulrush and darbha combination had the best NRC of 0.90, which is much more responsible for absorbing sound there in the crucial frequency spectrum of 500 to 2000 Hz. Natural fiber fillers of this type are excellent sound absorbers and are found in a variety of settings, including classrooms, recording studios, and theatres [118,119,120]. Table 2 shows the sound absorption coefficient of various fiber-reinforced polymer composites along with the density or porosity of the composites, and the measuring frequency.

Hence, the natural fiber fillers of this type are excellent sound absorbers and are found in a variety of settings, including classrooms, recording studios, and theatres [151,152,153].

## 4. Summary and Conclusions

Moisture absorption and sound absorption properties of various polymer composites were reviewed. The necessity of natural-fiber-reinforced polymer composites in various applications has a wider scope these days, but their poor compatibility and water absorption behavior retards the application spectrum. It was stated in many of the studies that the moisture absorption of the natural fibers and their composites is due to the hydrophilic nature of the natural fibers when they come into contact with moisture content. The moisture absorption of natural fiber composites increases with the content of natural fiber and this deteriorates the strength of natural fiber composites by paving the way for the initiation of microcracks at the interfacial region of matrix and reinforcements. Moisture absorption is linearly proportional with time but saturates after reaching the saturation point beyond which the moisture absorption capability of the composites remains constant. As natural fibers absorb more moisture relatively, the use of synthetic matrix materials is encouraged for many instances. Various physical and chemical treatment methods help in retarding the moisture absorption capability of natural fiber polymer composites.

However, utilization of natural materials as acoustic insulators is booming these days in spite of various prevalent disadvantages with such natural materials, which includes poor flammability and thickness reduction. Solving these issues through the use of nanomaterials as hybrid reinforcements mitigates the aforesaid disadvantages and expands the scope of natural-fiber-reinforced composites in soundproofing applications. Natural fiber and particulate-based porous composite materials are widely used in many applications, including building layouts, automotive applications, marine and aviation applications, sound-insulating industrial cabinets, classroom environments, and home theatres. In order to continuously obtain good acoustical performance from the natural fiber polymer composites, it is necessary to address various physical parameters associated with the manufacturing of composites and the measuring of sound absorption, which is completely aligned with the environmental point of view. Such water- and sound-resistant polymer composites find their applications in various parts of marine, automobile, aeronautical, and sports applications.

## Figures and Tables

**Table 1:** Hygrothermal properties of natural fiber polymer composites.

S. No.	Reinforcements	Weight Fraction (%)	Matrix	Time of Immersion (h/days) and Immersion Temperature (°C)	Moisture Absorption (%)	References
1	Bamboo/jute/glass fibers	2.5/2.5/5	Polyester	144 h and 25 °C	23.23	[74]
2	Waste hemp fibers	10	Polybenzoxazine	90 h and 30 °C	7.05	[75]
3	Jute/basalt fibers	5/15	Epoxy	120 h and 40 °C	6.95	[76]
4	Roselle/sugar palm	5/5	Polyurethane	24 h and 25 °C	8.48	[77]
5	Jute fiber/rosewood and padauk wood dust	10/2.5	Epoxy	15 days and 32 °C	4.42	[78]
6	Flax/nano TiO_2_	10/1.5	Epoxy	30 h and 25 °C	1.2	[79]
7	Continuous bamboo fibers	20	Epoxy	4 h and 100 °C	19	[80]
8	Sugar palm/glass fiber	10/20	Thermoplastic Polyurethane	168 h and 25 °C	9.78	[81]
9	Waste corn husk flour	25	Polyurethane	30 days and 25 °C	9.5	[82]
10	Hemp/sisal fibers	15/15	Epoxy	42 days and 25 °C	11.6	[83]
11	Abaca fiber	25	Polypropylene	80 days and 50 °C	15.09	[84]
12	Wood flour	20	Polypropylene	96 h and 25 °C	1.09	[85]
13	Kenaf fiber	40	Polypropylene	24 h and 25 °C	1.05	[86]
14	Wood flour	35	Polypropylene	48 h and 25 °C	11.57	[87]
15	Olive stone flour	30	Polypropylene	48 h and 25 °C	9.55	[87]
16	Rice husk ash filler	40	Polypropylene	24 h and 25 °C	15.31	[88]
17	Luffa cylindria fiber	30	Polypropylene	960 h and 25 °C	28.4	[89]
18	Jute fiber	40	Polypropylene	18 h and 23 °C	21.5	[90]
19	Jute fiber	30	Epoxy	336 h and 25 °C	8	[91]
20	Alfa pulps filler	35	Low-density Polyethylene	480 h and 25 °C	25.71	[92]
21	Bamboo mat	25	Polyester	1440 h	50.31	[93]
22	Ijuk fiber	30	Polypropylene	480 h and 23 °C	5.22	[94]
23	Hemp/glass hybrid	15/20	Epoxy	3600 h	21.31	[95]
24	Jute/glass hybrid	10/30	Unsaturated Polyester	504	58.36	[96]
25	Sisal/banana hybrid	20/15	Epoxy	50	11.48	[97]
26	Coir/glass hybrid	15/15	Epoxy	1440	39.16	[98]
27	Short snake grass fiber	25	Isopthallic polyester	120 days and 60 °C	29.79	[99]
28	*Phoenix* sp. fiber	25	Epoxy	100 days and 30 °C	17.52	[100]
29	Calotropis gigantea fiber	15	Polyester	15 days and 70 °C	18.12	[101]
30	Calotropis gigantea fiber	20	Epoxy	72 h and 25 °C	17.44	[102]
31	Tindora tendril fiber filled with haritaki nanopowder	20/7.5	Epoxy	8 h and 30 °C	5.87	[103]

**Table 2:** Acoustic properties of natural fiber polymer composites.

S.No	Composite Material	Bulk density (kg/m^3^)/Porosity (%)	Measuring Frequency Range (Hz)	Sound Absorption Coefficient	References
1	Pineapple leaf fiber in epoxy matrix	-	>1000	0.9	[121]
2	Rubber crumbs waste fiber in polyester matrix	-	1000–6000	0.93	[122]
3	Kapok fiber in epoxy matrix	8.3 kg/m^3^	100–6300	0.98	[123]
4	Flax, ramie, and jute fibers in epoxy matrix	-	1000–10,000	0.88	[124]
5	Rice waste fiber in polyurethane foam matrix	8.35 kg/m^3^	400–6400	0.9	[125]
6	Sugarcane bagasse in epoxy matrix	10.26 kg/m^3^	0–1600	0.17	[126]
7	Date palm fiber powder in cement matrix	-	200–2000	0.78	[127]
8	Banana fiber in polyester matrix	88.4%	500–6000	0.97	[128]
9	Mineralized wood flour in epoxy matrix	-	2000	0.4	[129]
10	Ijuk fiber in polyurethane matrix	83.5%	3000–4500	0.9	[130]
11	Grass fiber in epoxy matrix	-	2000	0.98	[131]
12	Flax fibers in epoxy matrix	8.5 kg/m^3^	63–6300	0.9	[132]
13	Kapok fiber in epoxy matrix	20 kg/m^3^	125–4000	0.405	[133]
14	Cotton fabric in epoxy matrix	92%	3000–3500	0.92	[134]
15	Nonwoven cotton fiber in polyester matrix	-	125–3000	0.638	[135]
16	Betelnut fiber in polypropylene matrix	85%	6000	0.42	[136]
17	Hemp fiber in polyester matrix	141 kg/m^3^	1000–4500	0.58	[137]
18	Sugarcane bagasse in polyvinyl alcohol matrix	200.8 kg/m^3^	172–2000	0.75	[137]
19	Sisal fiber in polyvinyl alcohol matrix	214.7 kg/m^3^	172–2000	0.69	[138]
20	Rice husk ash in glue matrix	170 kg/m^3^	200–6400	0.81	[139]
21	Yucca gloriosa fiber in epoxy matrix	200 kg/m^3^	63–6300	0.95	[140]
22	Coarse wool with binding fibers	249.55 kg/m^3^	60–6300	0.84	[141]
23	Pineapple leaf fiber in epoxy matrix	117 kg/m^3^	500–4500	0.91	[142]
24	Corn husk fiber in epoxy matrix	92%	1600–3250	0.88	[143,144,145]
25	Kenaf fiber in polylactic acid matrix	82%	1450–1522	0.8	[144,145,146]
26	Polyurethane foam in Coffee grounds polyol	88%	150–4000	0.96	[145,146,147]
27	Sugarcane bagasse in urea formaldehyde resin matrix	78%	500–4000	0.75	[146,147,148,149]
28	Date palm fiber in lime matrix	81%	750–6300	0.55	[147,148,149]
29	Date palm branch powder filled urea formaldehyde	-	800–1250	0.4	[148,149,150]
30	Broom fiber in epoxy matrix	73%	>1650	0.9	[149,150,151]
